# SLMAP3 isoform modulates cardiac gene expression and function

**DOI:** 10.1371/journal.pone.0214669

**Published:** 2019-04-01

**Authors:** Jana Mlynarova, Mayra Trentin-Sonoda, Fernanda Gaisler da Silva, Jennifer L. Major, Maysoon Salih, Marcela S. Carneiro-Ramos, Balwant S. Tuana

**Affiliations:** 1 Department of Cellular and Molecular Medicine, Faculty of Medicine, University of Ottawa, Ottawa, Canada; 2 Centro de Ciências Naturais e Humanas, Universidade Federal do ABC, Santo Andre, SP, Brazil; York University, CANADA

## Abstract

The sarcolemmal membrane associated proteins (SLMAPs) belong to the super family of tail anchored membrane proteins which serve diverse roles in biology including cell growth, protein trafficking and ion channel regulation. Mutations in human SLMAP have been linked to Brugada syndrome with putative deficits in trafficking of the sodium channel (Na_v_1.5) to the cell membrane resulting in aberrant electrical activity and heart function. Three main SLMAP isoforms (SLMAP1 (35 kDa), SLMAP2 (45 kDa), and SLMAP3 (91 kDa)) are expressed in myocardium but their precise role remains to be defined. Here we generated transgenic (Tg) mice with cardiac-specific expression of the SLMAP3 isoform during postnatal development which present with a significant decrease (20%) in fractional shortening and (11%) in cardiac output at 5 weeks of age. There was a lack of any notable cardiac remodeling (hypertrophy, fibrosis or fetal gene activation) in Tg hearts but the electrocardiogram indicated a significant increase (14%) in the PR interval and a decrease (43%) in the R amplitude. Western blot analysis indicated a selective and significant decrease (55%) in protein levels of Na_v_1.5 while 45% drop in its transcript levels were detectable by qRT-PCR. Significant decreases in the protein and transcript levels of the calcium transport system of the sarcoplasmic reticulum (SERCA2a/PLN) were also evident in Tg hearts. These data reveal a novel role for SLMAP3 in the selective regulation of important ion transport proteins at the level of gene expression and suggest that it may be a unique target in cardiovascular function and disease.

## Introduction

Ion channels and transporters located in sarcolemma (SL) and sarcoplasmic reticulum (SR) are key regulators of the electrical activity in cardiomyocytes and dictate their contraction and relaxation [1–3]. Depolarization is initiated by the sodium channels (Na_v_1.5) followed by entry of calcium through L-type calcium channels (Ca_v_1.2) which induces calcium release from SR through ryanodine receptors (RyR2). The increased intracellular Ca^2+^ activates the myofilaments leading to myocyte contraction [2,4]. Repolarization is initiated by potassium channels and coincides with the removal of cytosolic Ca^2+^ back in to the SR by the Ca^2+^-ATPase (SERCA2a) which is regulated by phospholamban (PLN) [1,2,4]. Other routes of Ca^2+^ removal include Na^+^/Ca^2+^ exchanger (NCX1)and the sarcolemmal Ca^2+^-ATPase. [1]. Alteration in the activity of these ion channels and transporters leads to abnormal electrical activity and changes in contraction/relaxation resulting in cardiac dysfunction [5–11].

Channelopathies are characterized by abnormal ion channel activity leading to organ dysfunction. The most prevalent cardiac channelopathies include long QT and Brugada syndrome [12,13]. Other less frequent channel disorders are known as congenital short QT syndrome, sinus node dysfunction, AV block, and progressive familial heart block. These phenotypes can occur separately or in combination and can lead to sudden cardiac death [12]. The channels that are commonly affected include calcium channels (Ca_v_1.2), potassium channels (KCN), and sodium channels (Na_v_1.5). Mutations in these ion channels and/or their interacting proteins can lead to changes in electrical activity resulting in arrhythmogenesis [13,14]. Mutations in Na_v_1.5 are implicated in multiple channelopathies [12,15]. Na_v_1.5 interacts with multiple factors affecting its structure, biophysical properties and trafficking to the sarcolemma. Mutations in Na_v_1.5 and its interacting proteins have been linked to the Brugada syndrome [16–18]. In this regard, mutations in the sarcolemmal membrane-associated protein (SLMAP) were linked to Brugada syndrome with associated deficits in Na_v_1.5 activity in a Japanese population [19]. A potential role for SLMAP in trafficking Na_v_1.5 to the sarcolemma was proposed although this needs to be fully interrogated. Thus, SLMAP may be categorized as protein that is important to support normal sodium channel function as genetic mutations lead to ion channelopathy [17].

The SLMAPs define a family of tail anchored membrane proteins which are generated by alternative splicing of the SLMAP gene [20]. At least 12 different SLMAP variants with three main isoforms designated SLMAP1, SLMAP2, and SLMAP3 are expressed in myocardium [21,22]. The shortest isoforms, SLMAP1 and SLMAP2, are highly expressed in cardiac muscle while the longest isoform SLMAP3 is ubiquitously expressed at low levels [22]. All SLMAP isoforms have a conserved central coiled-coil consisting of two tandem leucine zipper motifs responsible for dimerization. The C-terminus is a hydrophobic stretch of 21 amino acids which serves as a transmembrane (TM) anchor which can be alternatively spliced to target subcellular membranes of ER/SR, T-tubules/sarcolemma, mitochondria and perinuclear membrane in cardiomyocytes [23–25]. SLMAPs have been suggested to serve roles in organizing specialized membrane architecture involved in E-C coupling [24]. The SLMAP3 isoform carries an N-terminal extension that contains a forkhead associated (FHA) domain and an extended coiled-coil structure. The N-terminal domain in SLMAP3 targets the centrosome to impact cell growth and may play a role in signal transduction and gene programing to influence organ size [21,26,27].

Regulated levels of SLMAPs were shown to be critical in myoblast fusion and muscle development [25]. *In vivo* studies indicated that cardiac-specific overexpression of the SLMAP1 isoform in mice leads to cardiac remodeling with alteration of subcellular membrane structure, calcium cycling proteins and impaired SR Ca^2+^ transport which manifested as defective contractility at 28 weeks of age [28]. Aberrant expression of SLMAP was noted in animal models of diabetes and endothelial dysfunction while genetic variants were recently defined in diabetic retinopathy in humans [29,30] and mutations in SLMAP were linked to Brugada patients [18,19].

Here we investigate the role of SLMAP3 isoform in postnatal myocardium in mice with cardiac-specific gain of the full length SLMAP3 protein which encodes unique N-terminal sequences including a FHA domain distinct from the SLMAP1 isoform we examined previously (28). The data implies a novel role for the SLMAP3 isoform in the selective regulation of gene expression of ion channels and transporters *in vivo* with impact on cardiac biology.

## Materials and methods

### Transgenic mice with cardiac-specific expression of SLMAP3 and SLMAP1

Animals were handled and experiments were performed according to protocols reviewed and approved by the Animal Care Committee of the University of Ottawa that follows guidelines and regulations of the Canadian Council on Animal Care.

**Fig 1:**
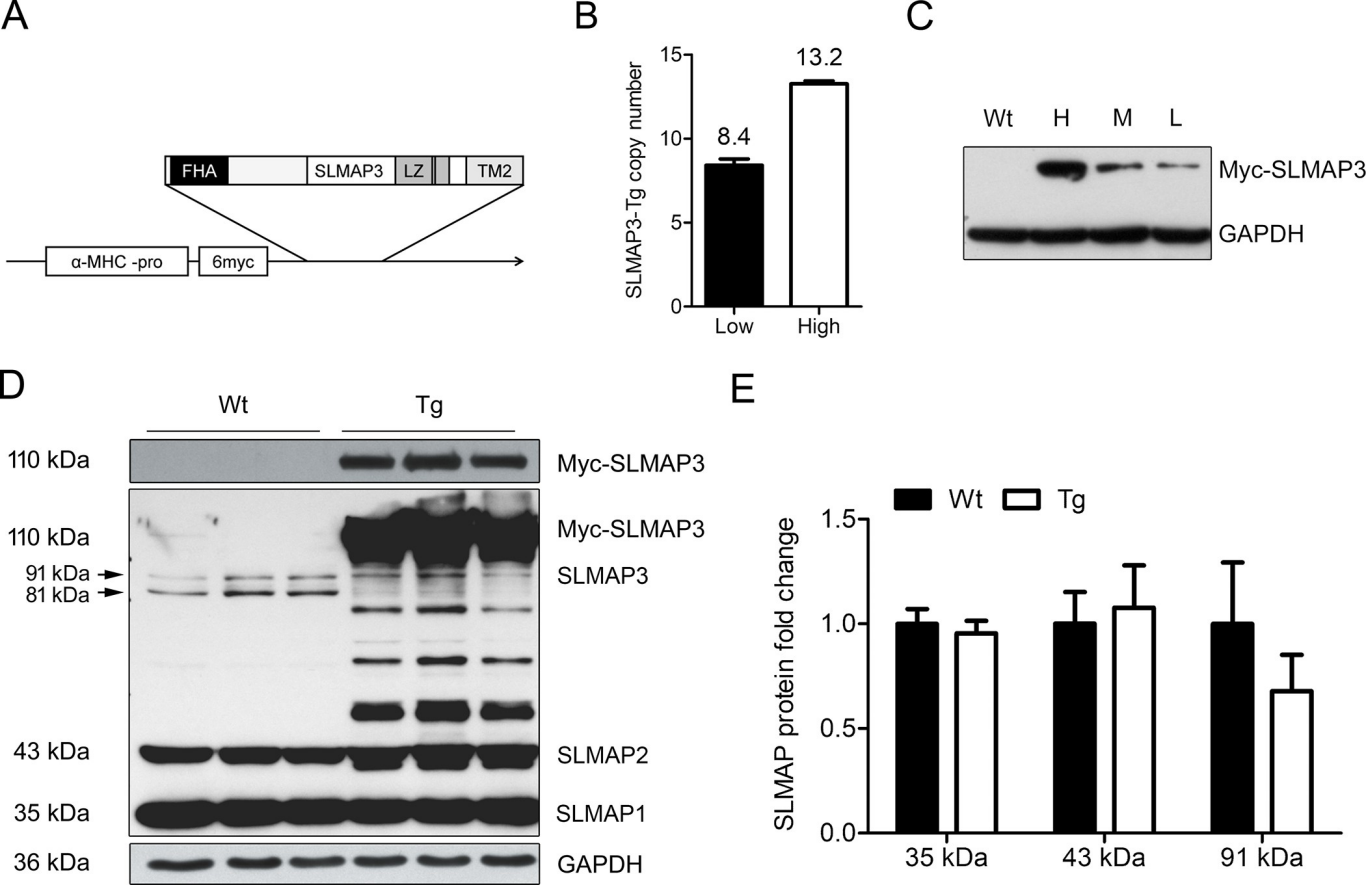
Postnatal expression of SLMAP3 in Tg mice. (A) Schematic representation of the SLMAP3 transgene construct comprising the forkhead associated domain (FHA), the leucine zipper coiled-coil region (LZ), and the transmembrane domain 2 (TM2) in frame with 6-myc tag, driven by α-MHC promoter. (B) Number of SLMAP3 transgene copies in hearts. DNA was isolated from hearts of mice and qRT-PCR was performed with appropriate primers to assess copies of Myc-SLMAP3 transgene. (C) SLMAP3 transgene protein expression in Tg mice. Western blot of heart lysate from Wt and Tg mice with anti-myc in high (H), moderate (M), and low (L) SLMAP3-six myc expression, GAPDH was used as loading control. (D) Endogenous SLMAP isoforms in Wt and Tg myocardium. Western blots of SLMAP isoforms in Wt and Tg mice at 5 weeks of age with anti-SLMAP (~35 kDa, ~43 kDa, ~81 kDa, ~91 kDa, and 110 kDa) and anti-myc antibodies (~110 kDa). SLMAP3 image was acquired with a longer exposure of the same membrane. (E) Quantification and fold change of protein expression levels of SLMAP1 (~35 kDa) and SLMAP2 (~43 kDa) isoforms in Tg mice compared to Wt age-matched littermates at 5 weeks of age; Wt corresponds to 1 (100%), n = 3.

Mice were generated as previously described [28]. SLMAP3 transgenic (Tg) animals were generated on B6D2F1 background by cardiac-targeted expression of full-length SLMAP3 sequence encompassing the leucine zipper coiled-coil region, forkhead associated domain and the transmembrane domain 2 (SLMAP3) driven by α-MHC promoter in frame with 6-myc tag. SLMAP3 construct (Fig 1A) was used to generate transgenic mice at the Toronto Centre of Phenogenomics, Toronto, CA. SLMAP1 transgenic (SLMAP1-Tg) animals were generated on B6C3F1 background by cardiac-targeted expression of SLMAP1 sequence encompassing the leucine zipper coiled-coil region and the transmembrane domain 2 (SLMAP1) driven by α-MHC promoter in frame with 6-myc tag [28]. Mice bearing the SLMAP3 or SLMAP1 transgene were identified by PCR analysis of genomic DNA and number of transgene copies were evaluated by qRT-PCR using the following primers: forward: 5′-TTAGCAAACCTCAGGCACCC-3′, reverse: 5′-CATAGCTTATCGATACCGTC-3′. We identified 3 lines, low, medium, and high expressers, based on Tg protein levels and number of SLMAP3 transgene copies (Fig 1B and 1C). SLMAP1-Tg lines were described previously [28]. We further studied the line with high expression of SLMAP3/SLMAP1 as there was no obvious phenotype. Tg mice were crossed/backcrossed and 3rd generation onwards up to 11th generation were used for analysis. Gender mixed population was used for the study.

### Survival analysis

Tg (n = 15) mice and their age-matched Wt (n = 16) littermates were daily checked for signs of distress and sudden death for 53 weeks. Remaining mice were then sacrificed and heart tissue was collected for further histological and biochemical analysis.

### Transthoracic echocardiography

Transthoracic echocardiography was performed using the Vevo 2100 high-resolution imaging system (VisualSonics, Toronto, ON, Canada) with a 30-MHz probe. Mice (14 Wt and 19 Tg) at 5 weeks of age were anesthetized with 2.5–3.5% isoflurane while acquiring images after 8–12 minutes from initial anesthesia. Level of used isoflurane was regulated based on heart rate of individual mice with a target of average 500 bpm with a maximum of 100 bpm range [31]. Left ventricular dimensions were acquired from six individual measurements in M-mode of the short axis view at the mid-ventricular level. LV end-diastolic and end-systolic diameter and volume, fractional shortening, ejection fraction, systolic and diastolic LV wall thickness, and LV mass were analysed with Vevo 1.6.0.6078 software. Cardiac output and stroke volume were calculated manually from acquired measurements.

### Electrocardiography

Mice (12 Wt and 9 Tg) were anesthetized with 2.5% isoflurane. 6-lead surface ECG was recorded after a 5 minute stabilization period of mice in the anesthetized state. ECG intervals and heart rate were analyzed manually from 6–18 most stable waves selected from a 2 minute recorded stream. Analysis was blind to the genotype. Intervals were defined as follows: PP duration from the beginning of the P wave to the end, where the P wave returns to the isoelectric line, PR segment from the end of the P wave to the beginning of R wave, PR interval from the beginning of P wave to the beginning of the R wave, QRS duration from the beginning of the R wave to the point where the negative S wave returns to the isoelectric line and QT interval from the beginning of the R wave to the point where the negative or positive T wave returns to the isoelectric line. Q wave was not visible. QT interval correction was based on Mitchell [32] using the following formula: QTc = QTo/(RRo/100)^1/2^. P, R, and S wave amplitudes were quantified as the distance between the peak of the wave and isoelectric line connecting the end of P wave and beginning of R wave. Electrocardiograms were recorded using IOX2.4.2.6 (EMKA Technologies, USA). RR interval, heart rate and wave amplitudes (P, R, and S) were analysed by ecgAUTO v2.5.1.18 software (EMKA Technologies, USA).

### Protein extraction and Western blot analysis

Cardiac tissue from 5 weeks old mice was used to isolate total protein lysate or microsomal fractions.

Total protein lysates were obtained by homogenizing heart tissue in RIPA buffer (1 mM EDTA, 150 mM NaCl, 1% NP-40, 0.25% Deoxycholic acid, 50 mM Tris pH 7.4) and centrifuged for 10 min at 12,000g. Supernatant was collected and stored at -80°C. 20 or 30 μg of protein lysate were separated on acrylamide gels and transferred to PVDF membrane using the wet transfer. PVDF membranes were probed with anti-SLMAP (1:750, #NBP1-81397, Novus Biologicals), anti-c-Myc (1:5000, #11667149001, Roche), anti-GAPDH (1:5000, # MA5-15738, ThermoFisher Scientific), and anti-α-Tubulin (1:20 000, #ab176560, Abcam). Protein expression was normalized to GAPDH, α-Tubulin or total protein quantified by stain-free technology (Bio-Rad). All densitometry analyses were performed in ImageLab 5.2 (Bio-Rad, USA).

For analysis of membrane proteins, heart tissue fractions were obtained as previously described [28]. Briefly, whole hearts were homogenized by a polytron homogenizer at medium speed with 4–5 four seconds long runs. Homogenate was centrifuged for 10 min at 1,600 g and supernatant was collected. Pellet was re-suspended and centrifuged at the same speed. Pooled supernatant corresponding to whole tissue lysate was centrifuged for 15 min at 14,000 g to pellet heavy SR fraction [33–36]. Supernatant was collected and centrifuged for 1 h at 45,000 g. Supernatant was collected as the cytosolic fraction. Pellet was re-suspended and centrifuged for 45 min at 48,000 g to pellet microsomal fraction. Supernatant was discarded and pellet re-suspended and stored at -80°C. 5 or 10 μg of protein fractions were separated on a 5–20% gradient acrylamide gel and transferred to PVDF membrane by wet transfer. Membranes were probed with following antibodies: anti-Na_v_1.5 (1:600, #ASC-005, Alomone Labs), anti-SERCA2a (1:1500, #MA3-919, ThermoFisher Scientific), anti-PLN (1:70 000, #A010-14, Badrilla), anti-phospho-PLN ser 16 (1:20 000, #A010-12, Badrilla), anti-calsequestrin (CSQ) (1:25 000, #PA1-913, BioReagents), anti-RyR2 (1:1500, #MA3-916, ThermoFisher Scientific), anti-phospho-RyR2 ser 2808 (1:10 000, #A010-30, Badrilla), and anti-Myc (1:5000, #11667149001, Roche). Calreticulin detected by anti-calreticulin (1:1000, #PA3-900, ThermoFisher Scientific) or total protein quantified using stain-free technology was used as loading control for protein quantification. Calreticulin was selected based on stability test prior to analysis of target proteins.

When using stain-free technology, stain-free gels and low fluorescence PVDF membranes (Bio-Rad) were used.

### Quantitative real-time PCR

RNA was extracted from cardiac tissue from age-matched Tg (n = 8) and Wt (n = 6) littermates at 5 weeks of age by RNeasy fibrous tissue mini kit (#74704, Qiagen). 2μg of RNA were reverse transcribed by High capacity cDNA reverse transcription kit (#4374966, ThermoFisher Scientific) following the manufacturer's guidelines. Equal amounts of cDNA (diluted 1:100) were used for qRT-PCR using primers listed in Table 1.

**Table 1:** Primers used for qRT-PCR.

Gene	Forward primer	Reverse primer
*Nppa* (ANP)	5‘-AGAGAGAGAAAGAAACCAGAGTGG-3‘	5‘-GTCTAGCAGGTTCTTGAAATCCAT-3‘
*Nppb* (BNP)	5‘-GCTGGAGCTGATAAGAGAAAAGTC-3‘	5‘-CAGGAGGTCTTCGTACAACAACTT-3‘
*Myh6* (α-MHC)	5‘-TCGTGCCTGATGACAAGGAG-3‘	5‘-TCGAACTTGGGTGGGTTCTG-3‘
*Myh7* (β-MHC)	5‘-CTTACTTGCTACCCTCAGGTGG-3‘	5‘-TGTCATCGGGCACAAAAACATC-3‘
*Scn5a* (Na_v_1.5)	5‘- AGAGCGAGTGTGAGTCCTTC-3‘	5‘- TGCTCTTCATACCCTCTGGAGT-3‘
*Slc8a1* (Ncx1)	5‘- CTTCAGAGCTGGTCGGTTTCT-3‘	5‘- GAGCTACCAGACGAAATCCCA-3‘
*Cacna1c* (Ca_v_1.2)	5‘- AACACTGAAAACGTGGCTGG-3‘	5‘-ACTTAACTGCTGCACGGCAT-3‘
*Atp2a2* (SERCA2a)	5‘-TAGCCAATGCAATCGTGGGT-3‘	5‘-ACACTTTGCCCATTTCAGGC-3‘
*Pln* (PLN)	5‘-TCAGGAGAGCCTCCACTATTGA-3‘	5‘- TTAAGCTGAGTTGGCATGTTGC-3‘
*Actb* (β-actin)	5‘-ACCCAGGCATTGCTGACAGGAT-3‘	5‘-CGCAGCTCAGTAACAGTCCGC-3‘

RNA from SLMAP1-Tg mouse hearts and Wt littermates at 5 weeks of age (n = 6) was extracted by TriPure Isolation Reagent (#11667165001, Roche). 2μg of RNA were reverse transcribed by SuperScript II Reverse Transcriptase (#18064–014, ThermoFisher Scientific) following the manufacturer's guidelines. Equal amounts of cDNA (diluted 1:50) were used for qRT-PCR using primers for *Scn5a* and β-actin listed in Table 1.

*Actb* (β-actin), *Rn18s* (18S rRNA) and *Gapdh* expression stability test conducted prior to analysis of target genes determined *Actb* as stable and thus was selected as a reference gene. Expression fold change of mRNA was calculated using the Pfaffl method [37] using reaction efficiencies defined by primer validation.

### Histological analysis

Hearts were fixed in 10% phosphate-buffered formalin for 24 hours and kept in 70% ethanol at 4°C until embedded into paraffin. Cardiac tissue sections were stained with hematoxylin and eosin (H&E) for visualisation of morphological changes and with Masson‘s Trichrome for detection of collagen deposits (fibrosis).

### Statistical analysis

Statistical analysis was performed using GraphPad Prism version 5.0 (GraphPad Software, La Jolla). Grubs test was used to determine possible outliers in echocardiography data. Wt and Tg mice were compared using the Student's t-test (all except some ECG data) or Mann-Whitney test (some ECG data) if data were not normally distributed. Normality of data distribution was tested by Shapiro-Wilk normality test. Data are presented as mean ± SEM. The level of statistical significance was selected as p< 0.05.

## Results

### Generation and characterization of transgenic mice with cardiac-specific expression of SLMAP3

Splicing of the SLMAP gene gives rise to 3 main isoforms SLMAP1, SLMAP2, and SLMAP3 in the myocardium. While SLMAP1 and SLMAP2 are cardiac specific, SLMAP3 isoform is ubiquitously expressed at low levels including the myocardium [22]. We generated a SLMAP3 cDNA construct encoding the full-length polypeptide (~91 kDa) that includes the forkhead associated (FHA) domain, leucine zipper coiled-coil region, and the transmembrane (TM) domain 2 in frame with a 6-myc tag and α-MHC promoter to drive expression specifically in the postnatal myocardium (Fig 1A). Mice were generated in a B6D2F1 genetic background and progeny screened for SLMAP3 transgene copies by qRT-PCR and protein levels of the endogenous (~91 kDa) and 6-myc SLMAP3 (~110 kDa), which were assessed by western blotting with anti-SLMAP or anti-Myc antibodies. Progeny were then characterized as low (8 copies), moderate or high (13 copies) expressers when screened for protein expression of 6-myc-SLMAP3 (Fig 1C) and transgene levels (Fig 1B). For moderate protein expressers transgene copies were not determined as they were not further bred. We used mice with high expression (13 copies of transgene) and corresponding levels of myc-SLMAP3 for further studies.

Western blot analysis of lysates from hearts of 5 weeks old Tg and Wt mice with anti-SLMAP (Fig 1D) detects bands at ~35 kDa, ~ 43 kDa, and ~91 kDa, previously described as SLMAP isoforms 1, 2, and 3 respectively [20,22]. The overexpressed (6-myc-SLMAP3) isoform is detected by both anti-SLMAP and anti-Myc antibodies at 110 kDa further discriminating the Tg mice from Wt. No differences in expression of the short SLMAP isoforms, SLMAP1 (~35 kDa) and SLMAP2 (~43 kDa), or endogenous SLMAP3 (~91 kDa) were observed between Wt and Tg mice (Fig 1D and 1E). Thus we concluded that overexpression of SLMAP3 in the heart does not affect the expression of the endogenous cardiac SLMAP isoforms.

Survival analysis of Tg (n = 15) mice and their Wt (n = 16) littermates up to the age of 53 weeks resulted in death of only 1 Wt mouse at 45 weeks of age (318 days). Furthermore, no distress or behavioral changes were noted in Tg. Thus our study indicates that high levels of SLMAP3 in the heart do not lead to any severe disease occurrence or premature death.

### Phenotyping SLMAP3 Tg myocardium

**Fig 2:**
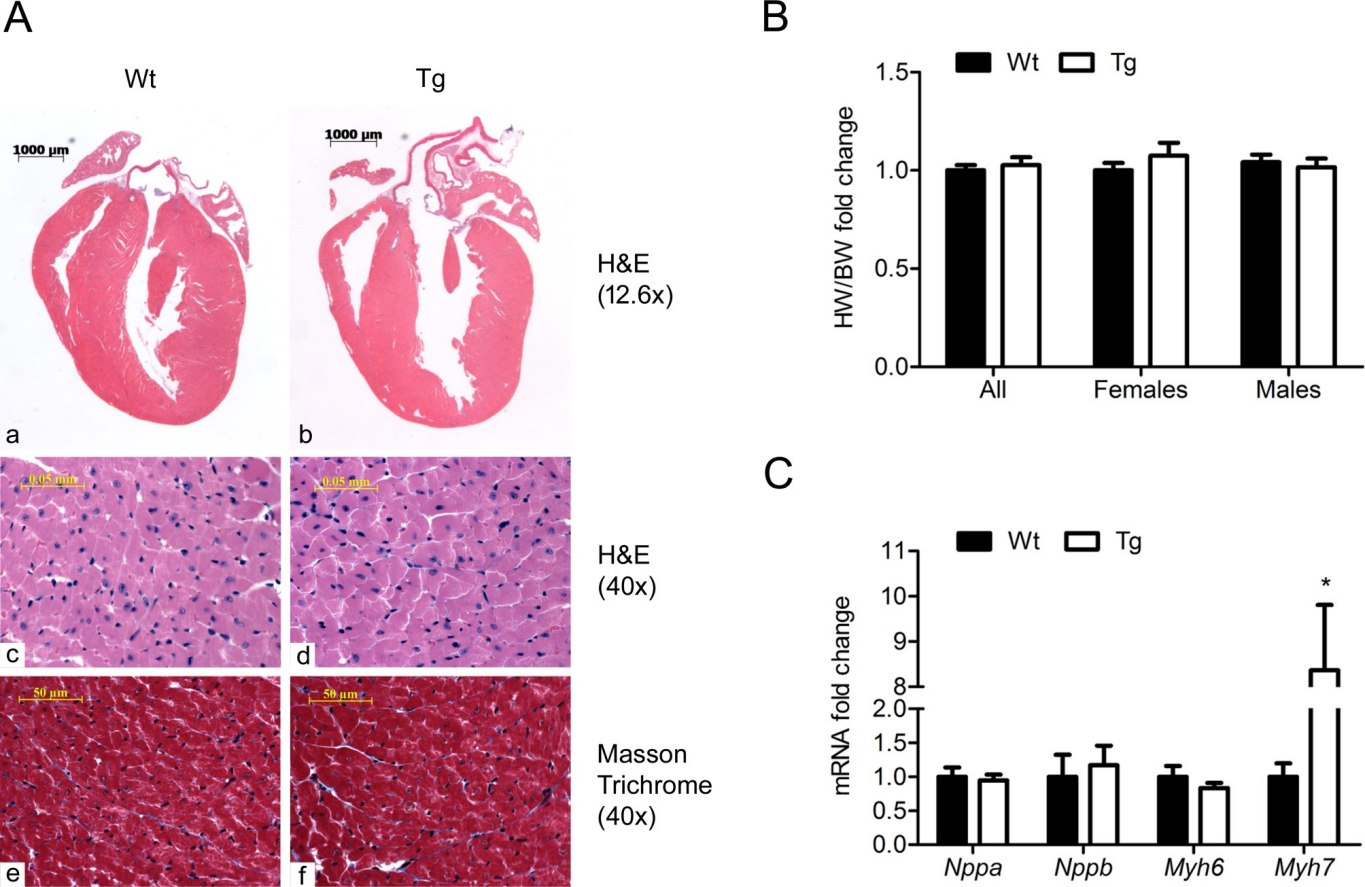
Histology and cardiac remodeling in SLMAP3 Tg mice. (A) H&E stained paraffin sections of the heart in Wt (a, c) and Tg (b, d) mice at 6 weeks of age. Masson’s Trichrome stained sections of Wt (e) and Tg (f) hearts at 6 weeks of age. Four chamber view sections of the heart were acquired at 12.6x magnification. Pictures at 40x magnification of the four chamber view are representative of random selections throughout the heart. (B) Heart weight (HW) corrected to body weight (BW) at 5 weeks of age acquired during necropsy. Values of mixed groups are represented as fold change of Tg compared to Wt mice. Sex specific groups are represented as fold change compared to Wt females. (C) qRT-PCR of fetal genes: *Nppa* (ANP), *Nppb* (BNP), *Myh6* (α-MHC), and *Myh7* (β-MHC) transcript levels at 5 weeks of age (6 Wt and 8 Tg); *p<0.05.

Cardiac morphology and molecular remodeling were evaluated in SLMAP3 Tg mice. Four chamber view of histology sections of the heart from six weeks old Tg and Wt mice with H&E staining indicates a difference in ventricular diameters (Fig 2A: a, b). Left ventricle size was further estimated by echocardiography (results shown in “Cardiac function in SLMAP3 Tg mice”). Higher magnification (40x) of these sections didn’t show any microscopic morphologic abnormalities (Fig 2A: c, d). Masson‘s Trichrome stained sections of the same hearts for collagen density (blue) did not show any obvious differences between Tg and Wt mice at higher magnification (40x) of the four chamber view (Fig 2A: e, f).

Heart weight (in grams) and body weight (in grams) were determined during necropsy of five weeks old Tg (n = 23) and Wt (n = 24) mice and heart weight-to-body weight ratio in a mixed sex population showed no change in Tg mice (0.0062 ± 0.0001 vs 0.0064 ± 0.0002 in Wt vs Tg). No difference in heart weight-to-body weight ratio was noted between Wt and Tg mice in females or males indicating there is no sex dependence (Fig 2B).

Fetal gene expression was analyzed by qRT-PCR of *Nppa* (ANP), *Nppb* (BNP), *Myh6* (α-MHC), and *Myh7* (β-MHC) in hearts from five weeks old Tg (n = 8) mice and age-matched Wt (n = 6) littermates (Fig 2C). Although *Nppa*, *Nppb*, or *Myh6* (mRNA) levels did not show any significant change in Tg mice, there was more then 8-fold increase in *Myh7* (mRNA) levels. All together, these data show no pronounced hypertrophy or fibrosis and no activation of the fetal gene program due to SLMAP3 expression, although a selective increase in *Myh7* transcripts was notable.

### Cardiac function in SLMAP3 Tg mice

**Fig 3:**
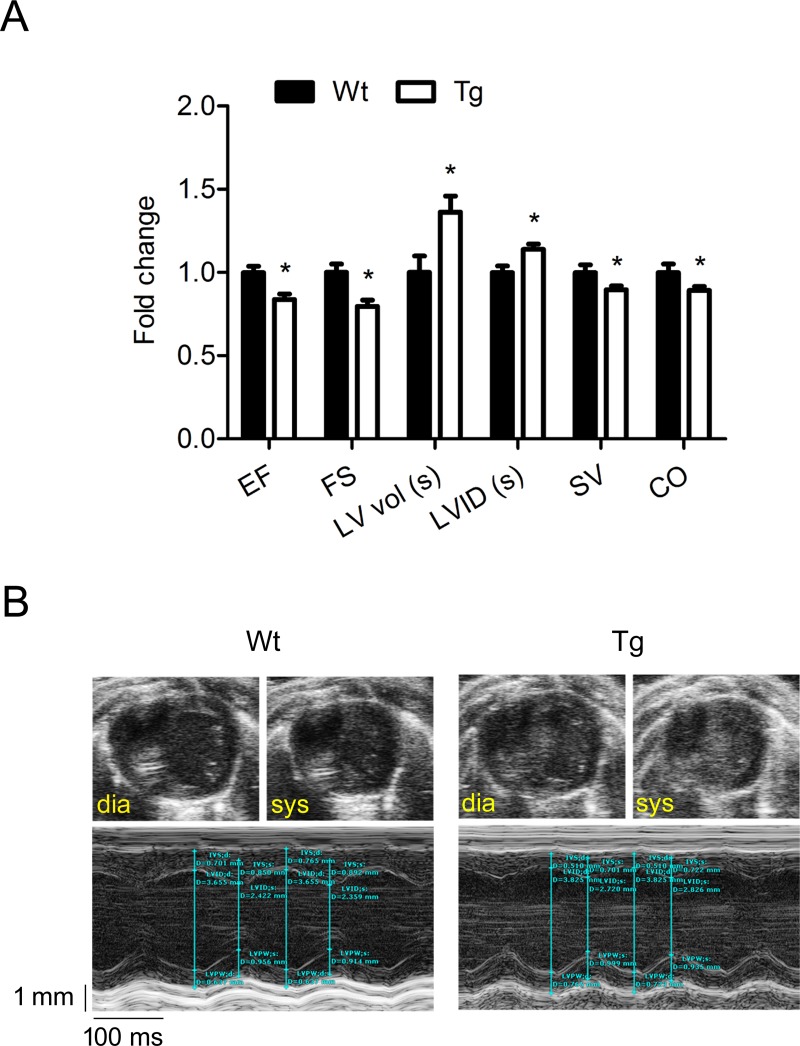
Function of myocardium in SLMAP3 Tg mice. (A) Heart performance and dimensions measured by transthoracic echocardiography in SLMAP3 Tg mice and Wt littermates at 5 weeks of age: ejection fraction (EF), fractional shortening (FS), end-systolic left ventricular volume (LV vol (s)), stroke volume (SV), cardiac output (CO), end-systolic left ventricular internal diameter (LVID (s)). (B) Representative echocardiograms of Wt and Tg mice showing end-diastole and end-systole in B-mode of the short axis with corresponding measurements in M-mode. *p<0.05 Tg compared to age matched Wt littermates.

Cardiac function was examined by transthoracic echocardiography in five week old mice (Table 2). Ejection fraction (EF) and fractional shortening (FS) were significantly decreased by 16% (62.7 ± 2.4 vs 52.5 ± 2.1% in Wt vs Tg, p<0.05) and 20% respectively (33.6 ± 1.7 vs 26.8 ± 1.3% in Wt vs Tg, p<0.05) in Tg mice compared with their Wt littermates. A significant increase in left ventricular volume and diameter in systole by 36% (22.9 ± 2.3 vs 31.2 ± 2.2 μl in Wt vs Tg, p<0.05) and 14% (2.5 ± 0.1 vs 2.8 ± 0.1 mm in Wt vs Tg, p<0.05) respectively was observed. Cardiac dysfunction was also evidenced by a significant decrease in stroke volume and cardiac output in Tg mice by 10% (37.3 ± 1.7 vs 33.4 ± 0.9 μl in Wt vs Tg, p<0.05) and 11% (17.8 ± 0.9 vs 15.9 ± 0.4 ml/min in Wt vs Tg, p<0.05) respectively (Fig 3). A slight increase in left ventricular posterior wall thickness at the end of diastole by 10% (p = 0.08) in Tg mice was noted. These results indicate an early onset of a significant systolic dysfunction of the heart in SLMAP3 Tg mice with increased left ventricular end-systolic volume.

**Table 2:** Echocardiography of SLMAP3 Tg mice.

Echocardiography parameter	WT (n = 14)	Tg (n = 19)	Change (%)	p value
EF (%)	62.7 ± 2.4	52.5 ± 2.1*	**16**	p = 0.003
FS (%)	33.6 ± 1.7	26.8 ± 1.3*	**20**	p = 0.003
LV mass/BW (mg/g)	3.4 ± 0.2	3.6 ± 0.1	7	P = 0.199
LV vol (d) (μl)	60.2 ± 3.1	64.7 ± 2.0	7	P = 0.218
LV vol (s) (μl)	22.9 ± 2.3	31.2 ± 2.2*	**36**	p = 0.015
Stroke volume (μl)	37.3 ± 1.7	33.4 ± 0.9*	**10**	p = 0.047
Cardiac output (ml/min)	17.8 ± 0.9	15.9 ± 0.4*	**11**	p = 0.043
LVID (d) (mm)	3.7 ± 0.1	3.9 ± 0.0	3	p = 0.179
LVID (s) (mm)	2.5 ± 0.1	2.8 ± 0.1*	**14**	p = 0.011
IVS (d) (mm)	0.7 ± 0.0	0.7 ± 0.0	3	p = 0.577
IVS (s) (mm)	0.9 ± 0.0	0.9 ± 0.0	2	p = 0.723
LVPW (d) (mm)	0.6 ± 0.0	0.7 ± 0.0	10	p = 0.082
LVPW (s) (mm)	0.9 ± 0.0	0.9 ± 0.0	1	p = 0.810

Left ventricular performance and dimensions measured by transthoracic echocardiography in 5 weeks old SLMAP3-Tg (n = 19) mice and their age-matched (Wt) littermates (n = 14). Measurements were acquired in M-Mode of the short axis view. EF = ejection fraction, FS = fractional shortening, LV mass/BW = left ventricular mass corrected to body weight, LV vol = left ventricular volume, LVID = left ventricular internal diameter, IVS = interventricular septal thickness, LVPW = left ventricular posterior wall thickness, (s) = systole, (d) = diastole. Values are presented as mean ± SEM

*p<0.05

### Electrophysiology of the heart in SLMAP3 Tg mice

**Fig 4:**
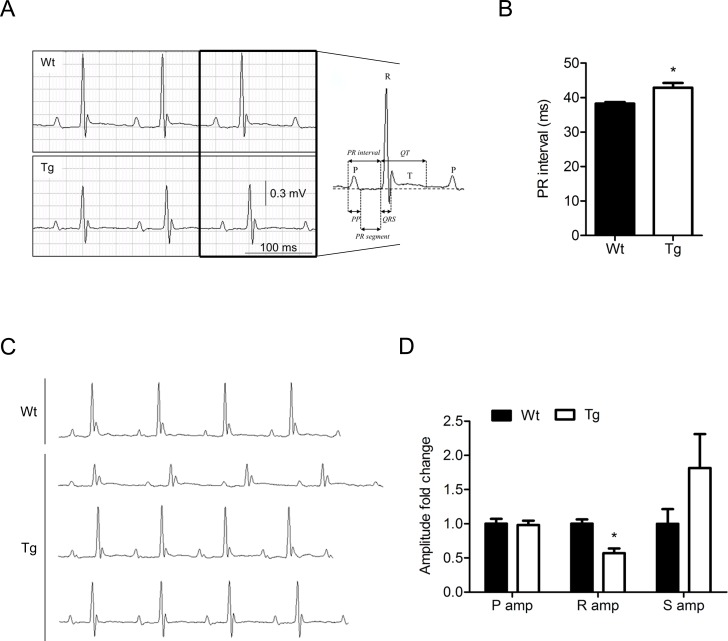
Electrical properties of myocardium in SLMAP3 Tg mice. (A) Representative electrocardiograms of lead II acquired by surface 6-lead ECG and definition of measured intervals in Wt (n = 11) and Tg (n = 8) mice at 5 weeks of age. (B) Quantification of PR interval in Wt and Tg mice measured in lead II. (C) Electrocardiograms acquired in Wt and Tg mice with QRS complexes different from the representative tracings shown in panel A. (D) Quantification of P, R, and S wave amplitudes in Wt and Tg mice measured in lead II. *p<0.05 Tg compared to age matched Wt littermates.

To determine if SLMAP3 affects the electrical properties of the heart, 6-lead surface electrocardiography was performed on five weeks old Wt (n = 12) and Tg (n = 9) mice (Table 3). A significant increase in the PR interval (Fig 4B) of Tg mice by 12% (38.24 ± 0.44 vs 42.84 ± 1.40 ms in Wt vs Tg, p<0.05) was observed. Other measured intervals- P wave duration, PR segment, QRS duration, and QTc interval- did not show any significant changes in SLMAP3 Tg mice. No difference in heart rate or RR interval was observed. On the other hand, lower R wave amplitude of the QRS complex and a more negative S wave were observed in 50% of Tg mice compared to Wt (Fig 4A). Two Tg mice appeared to have lower R wave amplitude with a small S wave, 1 Tg mouse presented an ECG curve similar to Wt with no development of S wave under the isolelectric line and 1 Tg mouse showed a R wave amplitude similar to Wt but larger S wave. 1 Tg mouse was excluded for excessive noise in the ECG signal and 1 Wt mouse was excluded for aberrant ECG recording with an R wave notch and no S wave. 1 Wt mouse presented an ECG curve without an S wave developing under the isoelectric line (Fig 4C). Quantification of P, R, and S wave amplitudes revealed a 43% lower R wave amplitude in Tg mice compared to their Wt littermates (0.673 ± 0.042 vs 0.383 ± 0.047 mV in Wt vs Tg). Both P and S amplitudes were comparable in both genotypes (Fig 4D). Together the elongated PR, depressed R, and more negative S wave observed in Tg mice suggest a dysfunctional atrioventricular conduction system which could result in a difference of conduction in the ventricles.

**Table 3:** Electrocardiography of SLMAP3 Tg mice.

Electrocardiography parameter	Wt (n = 11)	Tg (n = 8)	Change (%)	p value
RR (ms)	124.4 ± 2.8	126.9 ± 3.9	2	p = 0.606
HR (bpm)	484.6 ± 10.4	476.3 ± 15.3	2	p = 0.644
PP duration (ms)	12.94 ± 1.29	15.96 ± 1.83	23	p = 0.181
PR segment (ms)	25.29 ± 1.16	26.88 ± 1.98	6	p = 0.386
PR interval (ms)	38.24 ± 0.44	42.84 ± 1.40*	**14**	p = 0.002
QRS duration (ms)	9.90 ± 0.36	10.75 ± 0.29	9	p = 0.105
QTc interval (ms)	41.75 ± 3.92	30.85 ± 5.03	26	p = 0.127
P amplitude (mV)	0.089 ± 0.006	0.088 ± 0.006	1	p = 0.853
R amplitude (mV)	0.673 ± 0.042	0.383 ± 0.047*	**43**	p = 0.002
S amplitude (mV)	-0.062 ± 0.013	-0.112 ± 0.031	81	p = 0.113

Heart conduction system performance measured by surface 6-lead electrocardiography in 5 weeks old SLMAP3-Tg (n = 8) mice and their age-matched (Wt) littermates (n = 11). Intervals were calculated manually and amplitudes were calculated by ecgAUTO software in lead II in all mice. Data is presented as mean ± SEM

*p<0.05

### SLMAP3 regulates Na_v_1.5, SERCA2a, and PLN protein and transcript levels

**Fig 5:**
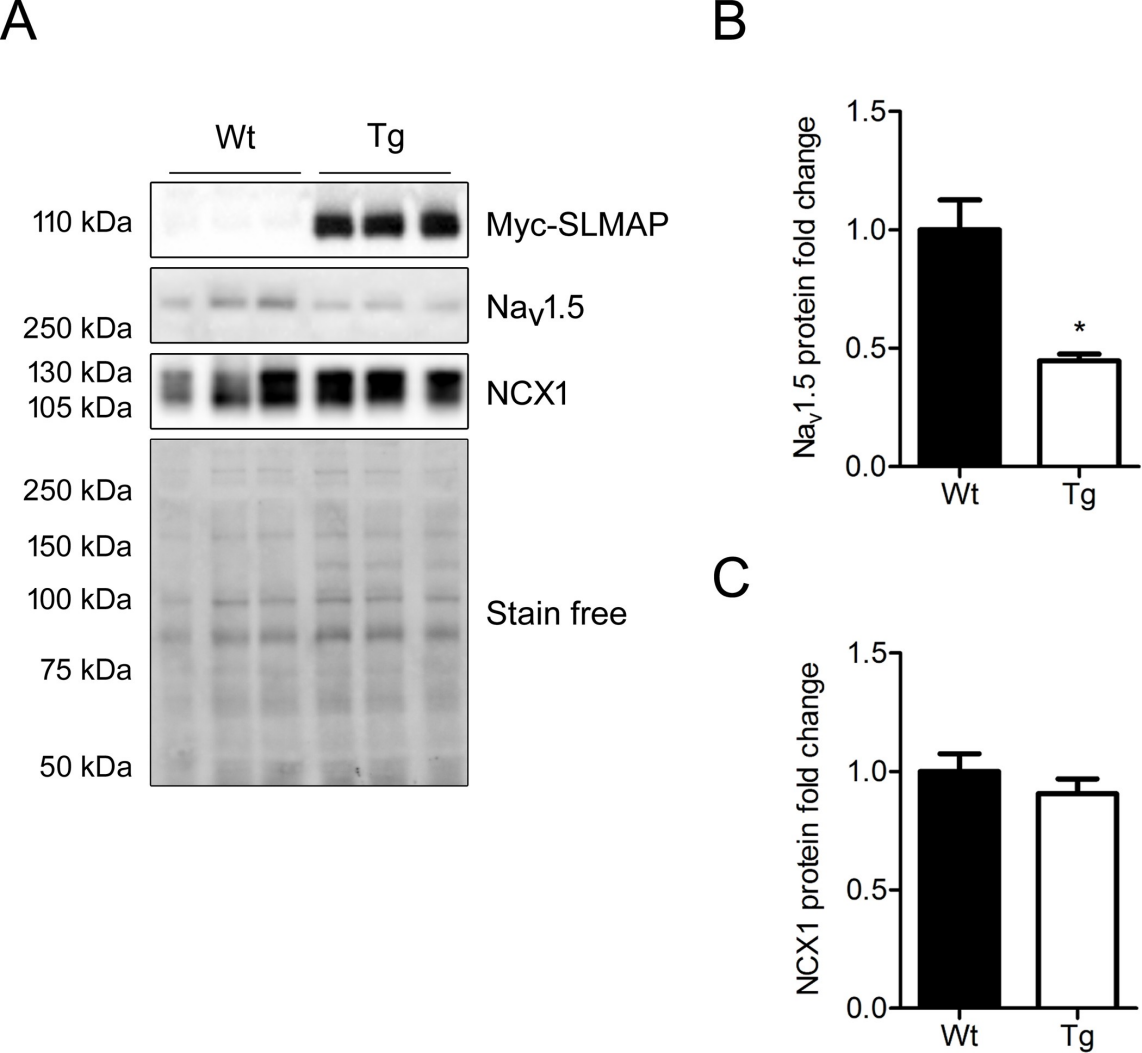
Na_v_1.5 and NCX1 expression in membrane fractions from Tg myocardium. (A) Western blot with anti-Na_v_1.5 and anti-NCX1 protein expression in the heavy SR fractions from 5 weeks old hearts from Wt and Tg mice (n = 3). (B) Protein quantification of Na_v_1.5 in heavy SR fraction. (C) Protein quantification of NCX1 in heavy SR fraction; Na_v_1.5 and NCX1 protein quantifications were normalized to total protein with stain-free and are presented as fold change where Wt = 1 (100%); quantified range of total protein is presented; Tg SLMAP was detected by anti-myc (Myc-SLMAP3).

Since SLMAP3-Tg mice presented with a decreased cardiac function and conduction deficits, we looked for changes in sodium and calcium handling proteins that may account for this phenotype. Western blots with anti-Na_v_1.5 detected a 250 kDa polypeptide in heavy SR membrane fractions (14 000g pellet), which are known to be enriched in T-tubules/SR terminal cisternae. This polypeptide was 55% (1.00 ± 0.13 vs 0.45 ± 0.03 in Wt vs Tg, p<0.05) decreased in Tg mice (Fig 5A, and quantified Fig 5B). Western blot analysis with anti-NCX1 (sodium/calcium exchanger) revealed no change in the 110 kDa NCX1 protein levels in the heavy SR membrane fractions from SLMAP3-Tg myocardium compared to Wt (Fig 5A and quantified Fig 5C).

**Fig 6:**
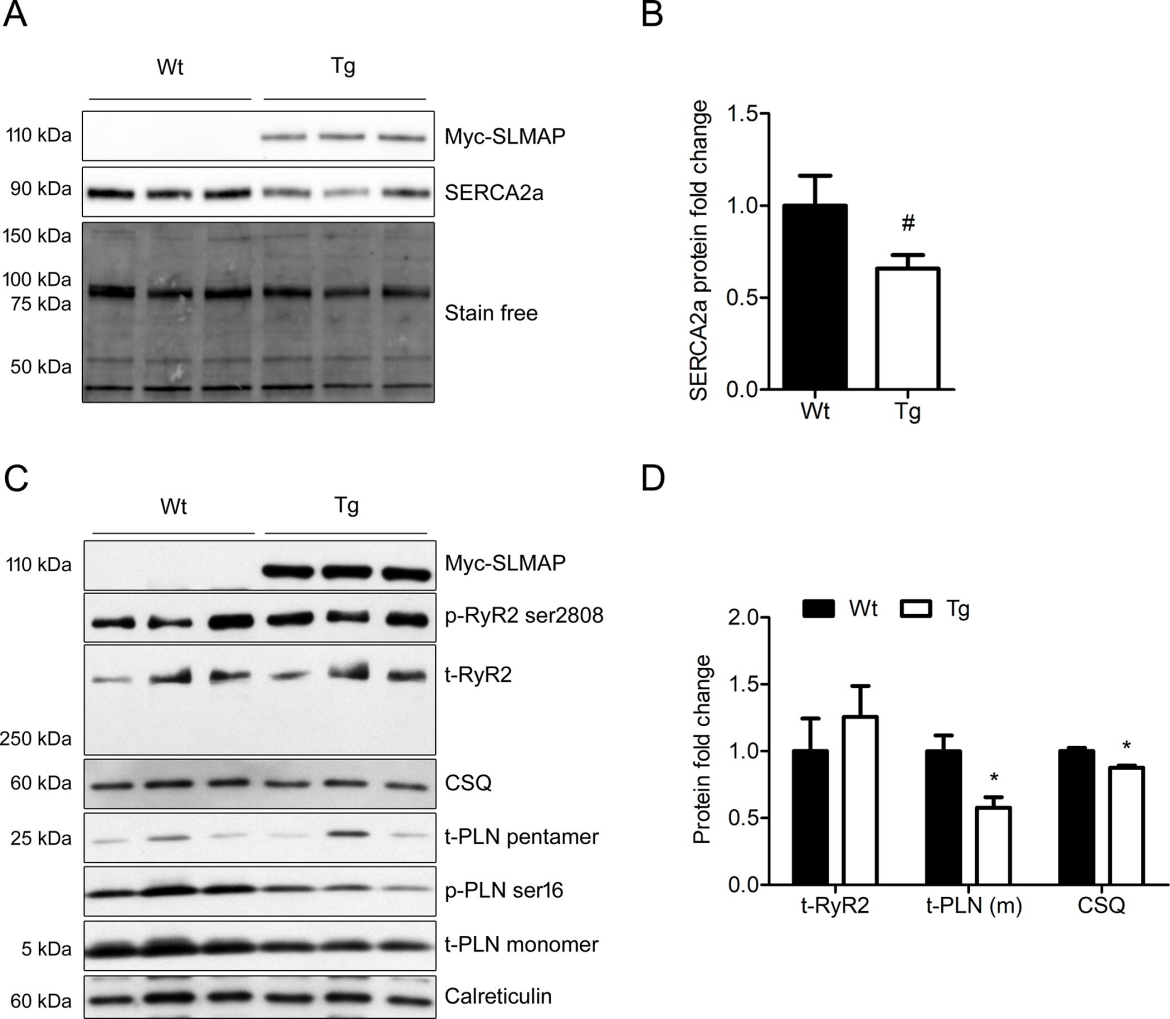
Expression of calcium handling proteins and their phosphorylation in microsomes. (A) Western blot analysis of SERCA2a protein expression in microsomal fractions and total protein acquired by stain-free. (B) Quantification of SERCA2a in microsomal fractions normalized to total protein assessed by stain free technology in Wt (n = 4) and Tg (n = 7) mice. (C) Western blots for total ryanodine receptor 2 (t-RyR2), it’s phosphorylation on serine 2808 (p-RyR2 ser 2808), calsequestrin (CSQ), total phospholamban (t-PLN) and it’s phosphorylation on serine 16 (p-PLN ser16) in microsomal fractions from 5 weeks old Tg and Wt mice hearts. Anti-calreticulin was used as loading control. Tg SLMAP3 was detected by anti-myc (Myc-SLMAP3). (D) Quantification of RyR2, CSQ, and PLN (monomeric) protein expression in Wt and Tg mice. *p<0.05, ^#^p = 0.053, n (Wt) = 5, n (Tg) = 4.

Western blot analysis of microsomal fractions revealed that SERCA2a (Fig 6A and 6B), PLN (monomeric), and calsequestrin (CSQ) (Fig 6C and 6D) are downregulated at the protein level by 34% (1.00 ± 0.16 vs 0.66 ± 0.07 in Wt vs Tg,p = 0.053), 42% (1.00 ± 0.12 vs 0.58 ± 0.08 in Wt vs Tg, p<0.05), and 12% (1.00 ± 0.02 vs 0.88 ± 0.02 in Wt vs Tg, p<0.05) respectively in SLMAP3-Tg myocardium compared to their Wt littermates. In contrast, western blotting with anti-RyR2 indicated it was unchanged in SLMAP3-Tg myocardium (Fig 6C and 6D) and anti-phospho antibodies revealed that phosphorylation of both RyR2 (ser2808) and PLN (ser16) remained unchanged in Tg mice compared to Wt.

**Fig 7:**
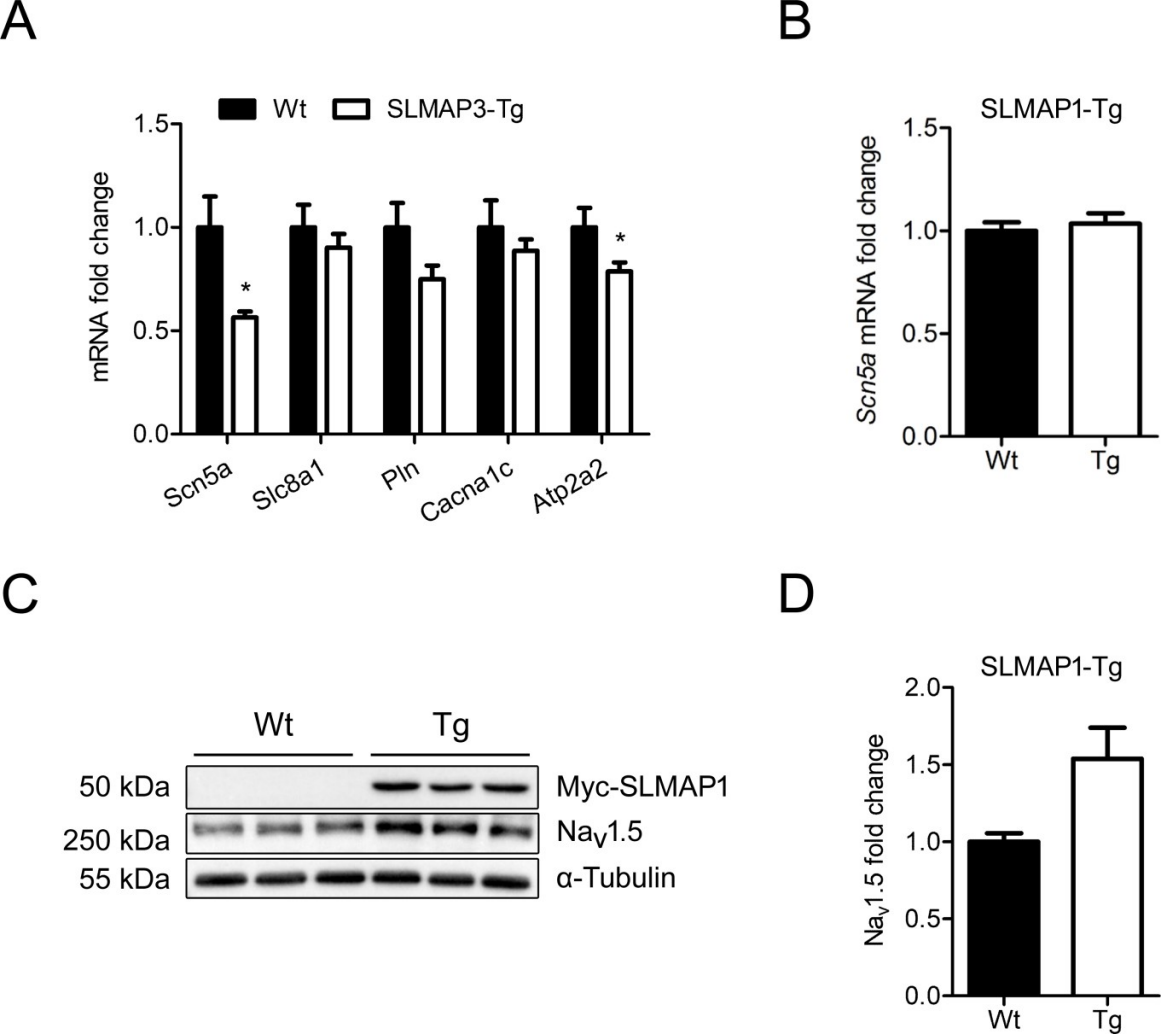
mRNA expression of sodium and calcium handling proteins in SLMAP3 and SLMAP1 Tg hearts. (A) mRNA levels of *Scn5a* (Na_v_1.5) sodium channel, *Slc8a1* (Ncx1) sodium calcium exchanger, *Pln*, *Cacna1c* (Ca_v_1.2), and *Atp2a2* (SERCA2a) assessed by qRT-PCR in hearts from 5 weeks old Wt (n = 6) and SLMAP3 Tg (n = 8) mouse hearts; (B) mRNA levels of *Scn5a* in 5 weeks old Wt and SLMAP1-Tg mouse hearts (n = 6). (C) Western blot of Na_v_1.5 and Tg SLMAP (detected by anti-myc, Myc-SLMAP1) in SLMAP1-Tg and Wt mouse hearts. (D) Quantification of Na_v_1.5 protein level normalized to α-Tubulin in SLMAP1-Tg and Wt mouse hearts (n = 3). Data are presented as fold change where Wt = 1 (100%), *p<0.05.

To determine if transcript levels of ion channels and transporters were being affected by SLMAP3, qRT-PCR was performed on RNA isolated from Tg hearts and Wt littermates (Fig 7A). Significant reductions in *Scn5a* (Na_v_1.5, mRNA) (44%,1.00 ± 0.15 vs 0.56 ± 0.03 in Wt vs Tg, p<0.05), *Atp2a2* (SERCA2a, mRNA) (21%,1.00 ± 0.09 vs 0.79 ± 0.04 in Wt vs Tg, p<0.05), and *Pln* (mRNA) (25%,1.00 ± 0.12 vs 0.75 ± 0.07 in Wt vs Tg, p = 0.07) levels were observed in SLMAP3 Tg mice. Similar to our observations on protein expression, no changes in *Slc8a1* (Ncx1, mRNA) or L-type calcium channel *Cacna1c* (Ca_v_1.2, mRNA) levels were observed in SLMAP 3 Tg hearts (Fig 7A).

In our previous study, the expression of the SLMAP1 isoform in postnatal myocardium also led to changes in electrical properties (QTc) and contractile defects which were manifested in much older (28 weeks of age) mice [28]. While changes in calcium handling proteins of the SR were evident, we did not examine effects on Na_v_1.5 expression in these Tg hearts. In the current study, we found no change in *Scn5a* (mRNA) transcript (Fig 7B) while Western blots indicate a slight increase in protein levels in SLMAP1-Tg myocardium (p = 0.063) (Fig 7C and 7D).

## Discussion

SLMAP belongs to a superfamily of tail anchored proteins which are involved in diverse functions including vesicle trafficking, membrane fusion, neurotransmitter release and ion channel regulation [20,38–41]. Mutations in human SLMAP have been shown to lead to Brugada syndrome due to potential defective trafficking of the alpha 1 subunit (Na_v_1.5) of the sodium channel to the sarcolemma although this needs to be confirmed [19]. Our data here reveal that increased SLMAP3 (91kDa) isoform levels in the postnatal myocardium *in vivo* can specifically impact gene and protein expression of the sodium channel (Na_v_1.5) and calcium transport system (SERCA2a/PLN) of the SR which may potentially explain the early changes in electrical activity and cardiac dysfunction noted in transgenic mice. Changes in the PR interval and a decrease in systolic function due to the gain of the SLMAP3 protein were evident in postnatal myocardium as early as 5 weeks and persisted up to 1 year without any death. A decrease in cardiac output was notable with a significant decline in fractional shortening, stroke volume with increased end systolic volume in Tg mice in absence of any hypertrophy or overt cardiac remodeling.

The electrocardiogram indicated changes in the PR interval which may be related to the decrease in the Na_v_1.5 protein in the Tg myocardium. Changes in PR interval have been linked to the aberrant expression of Na_v_1.5 (*SCN5A)* and Na_v_1.8 (*SCN10A*) in GWAS studies [42–48]. SLMAP3 levels appear to specifically impact this parameter of the ECG in SLMAP3 transgenic myocardium. It is also notable that the SLMAP gene resides at the 3p22.2 locus which correlates with association studies of Na_v_1.5 and Na_v_1.8 with the PR interval at this locus [22,49]. Thus our observation here that SLMAP3 can impact gene expression of Na_v_1.5 and the PR interval may be of significance in atrioventricular conduction and susceptibility to arrhythmia in humans [50]. It is known that loss-of-function mutations in Na_v_1.5 are associated with heart conduction disorders such as Brugada syndrome, sick sinus syndrome, atrial fibrillation and dilated cardiomyopathy [15]. Defects are reflected in ECG by prolonged atrial and atrioventricular conduction parameters as P wave, PR and QRS intervals [7,15]. Defective splicing of Na_v_1.5 and its expression has also been noted to lead to an increased PR interval, conduction defects and arrhythmia in myotonic dystrophy [51]. While Papadatos et al. reported that heterozygous Scn5a^+/-^ mice with 50% decrease in the Na_v_1.5 gene and protein expression did not exhibit any obvious abnormalities or difference in heart weight compared to Wt mice but presented with a prolongation of P wave and PR interval with no change in QTc or AV block [52]. These data are consistent with our observations in the SLMAP3-Tg mice which show a 55% decrease in Na_v_1.5 protein expression and prolongation of PR interval without changes in QTc. Increased PR interval is noted in Brugada patients who carry mutation in Na_v_1.5 or the proteins associated with it (34) while a significant decrease in the R amplitude has been proposed to be an early predictor of progression to MI [53].

The depressed cardiac function noted in SLMAP3-Tg mice as early as 5 weeks of age is likely related to the decreased levels of proteins involved in calcium handeling by the SR. SERCA2a and PLN levels were significantly down regulated as was calsequestrin in Tg hearts. SERCA2a/PLN is the calcium uptake mechanism involved in transporting calcium into the SR while calsequestrin is involved in binding/storing it [54]. There was no change in the expression of the RyR2 (or its phosphorylation), which is involved in calcium release [1]. It appears that SLMAP3 levels can selectively modulate the level of proteins that impact calcium uptake and storage capacity of the SR which is known to critically impact calcium cycling and contratile function of the heart [55]. It is notable that SLMAP3 is also selective in its effects on the sarcolemmal components since it had no effect on the expression of the Na^+^/Ca^2+^ exchanger or the L-calcium channel Ca_v_1.2 but specifically impacted Na_v_1.5 levels. In this regard we did not see any changes in Na_v_1.5 levels in the SLMAP1 –Tg mice and a distinctively mild cardiac phenotype was notable at only 28 weeks of age in these mice (28).

SLMAP3 expression does not lead to any severe health issues or sudden death as seen by the survival of Tg mice up to 53 weeks of age and the functional deficit did not progres from that noted at 5 weeks. While the SLMAP3 Tg mice indicated lower cardiac output due to a systolic dysfunction and prolongation of PR interval as early as 5 weeks, no activation of the fetal gene program or adverse membrane remodeling was evident at any age with the exception of the upregulation of β-MHC gene. This phenotype is uniquely different from that we reported for the overexpression of the SLMAP1 (35kDa) isoform in postnatal myocardium which led to a prolonged QTc and a decrease in cardiac contractility at 28 weeks of age and activation of the fetal gene program with remarkable subcellular membrane remodeling with distinctive vacuolation of myocytes [28]. While deficits in the calcium handeling proteins of the SR were also evident in the SLMAP1-Tg hearts including a decrease in RyR2 channel protein, there was no decrease in the expression of Na_v_1.5 as noted here in the SLMAP3-Tg myocardium. Although SLMAP1 is by far the most abundant isoform in the heart [22], these results indicate that SLMAP3 isoform may serve a uniquely distinct role in terms of gene expression of Na_v_1.5 and SERCA2a/PLN.

SLMAP3 is the longest of the SLMAP isoforms and contains an N-terminal forkhead associated (FHA) domain which targets the nucleus to regulate mitosis [21,22]. The FHA domain containing proteins are known regulators of gene transcription [56,57] and thus it is plausible that SLMAP3 isoform may serve such functions in modulating gene expression of Na_v_1.5 and SERCA2a/PLN. In this regard, SLMAP3 via it`s FHA domain has recently been shown to bind MST1/2, which is a key kinase involved in transcriptional signaling and organ growth [26,58,59]. How SLMAP3 integrates with the transcriptional programing of gene activity remains to be investigated but our data suggest that it may play a significant role in the expression of key membrane components involved in cardiac biology.

Ishikawa et al. linked missense mutations in SLMAP3 to Brugada patients in the Japanese population and provided evidence for a potential deficit in the trafficking of Na_v_1.5 protein to surface membrane and channel activity in co-transfection studies in HEK293 cells by the mutant SLMAP3 [19]. Our data here show that in postnatal myocardium in vivo, increased SLMAP3 protein can supress the mRNA levels of Na_v_1.5 accounting for the decrease in the channel protein in subcellular membranes potentially resulting in the increased PR interval. Thus regulated SLMAP3 levels may critically modulate the expression of Na_v_1.5 on the one hand and its appropriate trafficking on the other to impact normal cardiac electrophysiology and function. It is notable that the SLMAP1 isoform has been linked to endosomal trafficking of GLUT4 in myocardium implying that the different isoforms may serve distinct functions in trafficking [29,60]. SLMAP isoform specific gain and loss of function mouse models are being employed to decipher how SLMAPs modulate gene expression and protein targeting in myocardium.

In conclusion, we show that the gain of SLMAP3 protein in postnatal myocardium leads to a myocardial dysfunction without any adverse cardiac remodeling. The heart dysfunction is underlined by molecular changes that selectively impact the transcript and protein levels of Na_v_1.5 and SERCA2a/PLN *in vivo*. The data imply that SLMAP levels critically impact normal cardiac electrophysiology/function and with the genetic linkage in Brugada patients makes it a unique target in heart disease.

## References

[1] Bers DM. Cardiac excitation–contraction coupling. Nature. 2002;415: 198–205. 10.1038/415198a 11805843

[2] Nerbonne JM, Kass RS. Molecular Physiology of Cardiac Repolarization. Physiol Rev. 2005;85: 1205–1253. 10.1152/physrev.00002.2005 16183911

[3] Wehrens XHT, Lehnart SE, Marks AR. Intracellular calcium release and cardiac disease. Annu Rev Physiol. 2005;67: 69–98. 10.1146/annurev.physiol.67.040403.114521 15709953

[4] Grandi E M. Bers D. Models of the Ventricular Action Potential in Health and Disease In: Zipes D P., Jalife J, editors. Cardiac Electrophysiology: From Cell to Bedside. 6th ed Elsevier; 2013 pp. 319–330.

[5] Abriel H, Rougier J-S, Jalife J. Ion Channel Macromolecular Complexes in Cardiomyocytes: Roles in Sudden Cardiac Death. Circ Res. 2015;116: 1971–1988. 10.1161/CIRCRESAHA.116.305017 26044251 PMC4471480

[6] Adsit GS, Vaidyanathan R, Galler CM, Kyle JW, Makielski JC. Channelopathies from Mutations in the Cardiac Sodium Channel Protein Complex. J Mol Cell Cardiol. 2013;61: 34–43. 10.1016/j.yjmcc.2013.03.017 23557754 PMC3720718

[7] Amin AS, Asghari-Roodsari A, Tan HL. Cardiac sodium channelopathies. Pflüg Arch—Eur J Physiol. 2010;460: 223–237. 10.1007/s00424-009-0761-0 20091048 PMC2883928

[8] Belevych AE, Radwański PB, Carnes CA, Györke S. ‘Ryanopathy’: causes and manifestations of RyR2 dysfunction in heart failure. Cardiovasc Res. 2013;98: 240–247. 10.1093/cvr/cvt024 23408344 PMC3633158

[9] Goonasekera SA, Hammer K, Auger-Messier M, Bodi I, Chen X, Zhang H, et al Decreased cardiac L-type Ca2+ channel activity induces hypertrophy and heart failure in mice. J Clin Invest. 2012;122: 280–290. 10.1172/JCI58227 22133878 PMC3248289

[10] Lazzerini PE, Capecchi PL, Laghi-Pasini F, Boutjdir M. Autoimmune channelopathies as a novel mechanism in cardiac arrhythmias. Nat Rev Cardiol. 2017;14: 521–535. 10.1038/nrcardio.2017.61 28470179

[11] Schmitt N, Grunnet M, Olesen S-P. Cardiac Potassium Channel Subtypes: New Roles in Repolarization and Arrhythmia. Physiol Rev. 2014;94: 609–653. 10.1152/physrev.00022.2013 24692356

[12] Abriel H, Zaklyazminskaya EV. Cardiac channelopathies: Genetic and molecular mechanisms. Gene. 2013;517: 1–11. 10.1016/j.gene.2012.12.061 23266818

[13] Behere SP, Weindling SN. Inherited arrhythmias: The cardiac channelopathies. Ann Pediatr Cardiol. 2015;8: 210–220. 10.4103/0974-2069.164695 26556967 PMC4608198

[14] Napolitano C, Bloise R, Monteforte N, Priori SG. Sudden Cardiac Death and Genetic Ion Channelopathies: Long QT, Brugada, Short QT, Catecholaminergic Polymorphic Ventricular Tachycardia, and Idiopathic Ventricular Fibrillation. Circulation. 2012;125: 2027–2034. 10.1161/CIRCULATIONAHA.111.055947 22529064

[15] Remme CA, Bezzina CR. Sodium Channel (Dys)Function and Cardiac Arrhythmias. Cardiovasc Ther. 2010;28: 287–294. 10.1111/j.1755-5922.2010.00210.x 20645984

[16] Abriel H. Cardiac sodium channel Nav1.5 and interacting proteins: Physiology and pathophysiology. J Mol Cell Cardiol. 2010;48: 2–11. 10.1016/j.yjmcc.2009.08.025 19744495

[17] Kyle JW, Makielski JC. Diseases caused by mutations in Nav1.5 interacting proteins. Card Electrophysiol Clin. 2014;6: 797–809. 10.1016/j.ccep.2014.08.007 25395996 PMC4226528

[18] Sarquella-Brugada G, Campuzano O, Arbelo E, Brugada J, Brugada R. Brugada syndrome: clinical and genetic findings. Genet Med. 2016;18: 3–12. 10.1038/gim.2015.35 25905440

[19] Ishikawa T, Sato A, Marcou CA, Tester DJ, Ackerman MJ, Crotti L, et al A Novel Disease Gene for Brugada Syndrome: Sarcolemmal Membrane-Associated Protein Gene Mutations Impair Intracellular Trafficking of hNav1.5. Circ Arrhythm Electrophysiol. 2012;5: 1098–1107. 10.1161/CIRCEP.111.969972 23064965

[20] Wielowieyski PA, Sevinc S, Guzzo R, Salih M, Wigle JT, Tuana BS. Alternative Splicing, Expression, and Genomic Structure of the 3′ Region of the Gene Encoding the Sarcolemmal-associated Proteins (SLAPs) Defines a Novel Class of Coiled-coil Tail-anchored Membrane Proteins. J Biol Chem. 2000;275: 38474–38481. 10.1074/jbc.M007682200 10986292

[21] Guzzo RM, Sevinc S, Salih M, Tuana BS. A novel isoform of sarcolemmal membrane-associated protein (SLMAP) is a component of the microtubule organizing centre. J Cell Sci. 2004;117: 2271–2281. 10.1242/jcs.01079 15126628

[22] Wigle JT, Demchyshyn L, Pratt MAC, Staines WA, Salih M, Tuana BS. Molecular Cloning, Expression, and Chromosomal Assignment of Sarcolemmal-associated Proteins: A family of acidic amphipathic α-helical proteins associated with the membrane. J Biol Chem. 1997;272: 32384–32394. 10.1074/jbc.272.51.32384 9405447

[23] Byers JT, Guzzo RM, Salih M, Tuana BS. Hydrophobic profiles of the tail anchors in SLMAP dictate subcellular targeting. BMC Cell Biol. 2009;10: 48 10.1186/1471-2121-10-48 19538755 PMC2712456

[24] Guzzo RM, Salih M, Moore ED, Tuana BS. Molecular properties of cardiac tail-anchored membrane protein SLMAP are consistent with structural role in arrangement of excitation-contraction coupling apparatus. Am J Physiol-Heart Circ Physiol. 2005;288: H1810–H1819. 10.1152/ajpheart.01015.2004 15591093

[25] Guzzo RM, Wigle J, Salih M, Moore ED, Tuana BS. Regulated expression and temporal induction of the tail-anchored sarcolemmal-membrane-associated protein is critical for myoblast fusion. Biochem J. 2004;381: 599–608. 10.1042/BJ20031723 15086317 PMC1133868

[26] Couzens AL, Knight JDR, Kean MJ, Teo G, Weiss A, Dunham WH, et al Protein Interaction Network of the Mammalian Hippo Pathway Reveals Mechanisms of Kinase-Phosphatase Interactions. Sci Signal. 2013;6: rs15–rs15. 10.1126/scisignal.2004712 24255178

[27] Zheng Y, Liu B, Wang L, Lei H, Prieto KDP, Pan D. Homeostatic control of Hpo/MST kinase activity through autophosphorylation-dependent recruitment of the STRIPAK PP2A phosphatase complex. Cell Rep. 2017;21: 3612–3623. 10.1016/j.celrep.2017.11.076 29262338 PMC5741103

[28] Nader M, Westendorp B, Hawari O, Salih M, Stewart AFR, Leenen FHH, et al Tail-anchored membrane protein SLMAP is a novel regulator of cardiac function at the sarcoplasmic reticulum. Am J Physiol-Heart Circ Physiol. 2012;302: H1138–H1145. 10.1152/ajpheart.00872.2011 22180652

[29] Chen X, Ding H. Increased Expression of the Tail-Anchored Membrane Protein SLMAP in Adipose Tissue from Type 2 Tally Ho Diabetic Mice. Exp Diabetes Res. 2011;2011: 1–10. 10.1155/2011/421982 21785580 PMC3137969

[30] Ding H, Howarth AG, Pannirselvam M, Anderson TJ, Severson DL, Wiehler WB, et al Endothelial dysfunction in Type 2 diabetes correlates with deregulated expression of the tail-anchored membrane protein SLMAP. Am J Physiol-Heart Circ Physiol. 2005;289: H206–H211. 10.1152/ajpheart.00037.2005 15764684

[31] Gao S, Ho D, Vatner DE, Vatner SF. Echocardiography in Mice. Curr Protoc Mouse Biol. 2011;3 1: 71–83. 10.1002/9780470942390.mo100130 21743841 PMC3130310

[32] Mitchell GF, Jeron A, Koren G. Measurement of heart rate and Q-T interval in the conscious mouse. Am J Physiol-Heart Circ Physiol. 1998;274: H747–H751. 10.1152/ajpheart.1998.274.3.H747 9530184

[33] Goll DE, Young RB, Stromer MH. Separation of subcellular organelles by differential and density gradient centrifugation. Proceedings. 1974; 250–297.

[34] Graham J. Preparation of Crude Subcellular Fractions by Differential Centrifugation. Sci World J. 2002;2: 1638–1642. 10.1100/tsw.2002.851 12806153 PMC6009713

[35] Meissner G. Isolation and characterization of two types of sarcoplasmic reticulum vesicles. Biochim Biophys Acta BBA—Biomembr. 1975;389: 51–68. 10.1016/0005-2736(75)90385-5 124589

[36] Saito A, Seiler S, Chu A, Fleischer S. Preparation and morphology of sarcoplasmic reticulum terminal cisternae from rabbit skeletal muscle. J Cell Biol. 1984;99: 875–885. 6147356 10.1083/jcb.99.3.875 PMC2113387

[37] Pfaffl MW. A new mathematical model for relative quantification in real-time RT-PCR. Nucleic Acids Res. 2001;29: 45e – 45. 10.1093/nar/29.9.e45 PMC55695 11328886

[38] Borgese N, Colombo S, Pedrazzini E. The tale of tail-anchored proteins: coming from the cytosol and looking for a membrane. J Cell Biol. 2003;161: 1013–1019. 10.1083/jcb.200303069 12821639 PMC2173004

[39] Salaün C, James DJ, Greaves J, Chamberlain LH. Plasma membrane targeting of exocytic SNARE proteins. Biochim Biophys Acta BBA—Mol Cell Res. 2004;1693: 81–89. 10.1016/j.bbamcr.2004.05.008 15313010

[40] Teng FYH, Wang Y, Tang BL. The syntaxins. Genome Biol. 2001;2: reviews3012.1–reviews3012.7. 11737951 10.1186/gb-2001-2-11-reviews3012 PMC138984

[41] Wattenberg B, Lithgow T. Targeting of C-Terminal (Tail)-Anchored Proteins: Understanding how Cytoplasmic Activities are Anchored to Intracellular Membranes. Traffic. 2001;2: 66–71. 10.1034/j.1600-0854.2001.20108.x 11208169

[42] Butler AM, Yin X, Evans DS, Nalls MA, Smith EN, Tanaka T, et al Novel Loci Associated with PR Interval in a Genome-Wide Association Study of Ten African American Cohorts. Circ Cardiovasc Genet. 2012;5: 639–646. 10.1161/CIRCGENETICS.112.963991 23139255 PMC3560365

[43] Chambers JC, Zhao J, Terracciano CMN, Bezzina CR, Zhang W, Kaba R, et al Genetic variation in SCN10A influences cardiac conduction. Nat Genet. 2010;42: 149–152. 10.1038/ng.516 20062061

[44] Holm H, Gudbjartsson DF, Arnar DO, Thorleifsson G, Thorgeirsson G, Stefansdottir H, et al Several common variants modulate heart rate, PR interval and QRS duration. Nat Genet. 2010;42: 117–122. 10.1038/ng.511 20062063

[45] Pfeufer A, van Noord C, Marciante KD, Arking DE, Larson MG, Smith AV, et al Genome-wide association study of PR interval. Nat Genet. 2010;42: 153–159. 10.1038/ng.517 20062060 PMC2850197

[46] Smith JG, Lowe JK, Kovvali S, Maller JB, Salit J, Daly MJ, et al Genome-wide association study of electrocardiographic conduction measures in an isolated founder population: Kosrae. Heart Rhythm Off J Heart Rhythm Soc. 2009;6: 634–641. 10.1016/j.hrthm.2009.02.022 19389651 PMC2673462

[47] Smith JG, Magnani JW, Palmer C, Meng YA, Soliman EZ, Musani SK, et al Genome-Wide Association Studies of the PR Interval in African Americans. PLoS Genet. 2011;7: e1001304 10.1371/journal.pgen.1001304 21347284 PMC3037415

[48] Sotoodehnia N, Isaacs A, de Bakker PIW, Dörr M, Newton-Cheh C, Nolte IM, et al Common variants in 22 loci are associated with QRS duration and cardiac ventricular conduction. Nat Genet. 2010;42: 1068–1076. 10.1038/ng.716 21076409 PMC3338195

[49] Veerman CC, Wilde AAM, Lodder EM. The cardiac sodium channel gene SCN5A and its gene product NaV1.5: Role in physiology and pathophysiology. Gene. 2015;573: 177–187. 10.1016/j.gene.2015.08.062 26361848 PMC6636349

[50] Bidstrup S, Salling Olesen M, Hastrup Svendsen J, Bille Nielsen J. Role of PR-Interval In Predicting the Occurrence of Atrial Fibrillation. J Atr Fibrillation. 2013;6: 90–94. 10.4022/jafib.956 28496913 PMC5153137

[51] Freyermuth F, Rau F, Kokunai Y, Linke T, Sellier C, Nakamori M, et al Splicing misregulation of SCN5A contributes to cardiac-conduction delay and heart arrhythmia in myotonic dystrophy. Nat Commun. 2016;7: 11067 10.1038/ncomms11067 27063795 PMC4831019

[52] Papadatos GA, Wallerstein PMR, Head CEG, Ratcliff R, Brady PA, Benndorf K, et al Slowed conduction and ventricular tachycardia after targeted disruption of the cardiac sodium channel gene Scn5a. Proc Natl Acad Sci. 2002;99: 6210–6215. 10.1073/pnas.082121299 11972032 PMC122928

[53] Sun X, Cai J, Fan X, Han P, Xie Y, Chen J, et al Decreases in Electrocardiographic R-Wave Amplitude and QT Interval Predict Myocardial Ischemic Infarction in Rhesus Monkeys with Left Anterior Descending Artery Ligation. PLOS ONE. 2013;8: e71876 10.1371/journal.pone.0071876 23967258 PMC3742514

[54] Gwathmey JK, Yerevanian AI, Hajjar RJ. Cardiac gene therapy with SERCA2a: From bench to bedside. J Mol Cell Cardiol. 2011;50: 803–812. 10.1016/j.yjmcc.2010.11.011 21093451 PMC3075330

[55] Bers DM. Cardiac Sarcoplasmic Reticulum Calcium Leak: Basis and Roles in Cardiac Dysfunction. Annu Rev Physiol. 2014;76: 107–127. 10.1146/annurev-physiol-020911-153308 24245942

[56] Durocher D, Jackson SP. The FHA domain. FEBS Lett. 2002;513: 58–66. 10.1016/S0014-5793(01)03294-X 11911881

[57] Hofmann K, Bucher P. The FHA domain: a putative nuclear signaling domain found in protein kinases and transcription factors. Trends Biochem Sci. 1995;20: 347–349. 10.1016/S0968-0004(00)89072-6 7482699

[58] Bae SJ, Ni L, Osinski A, Tomchick DR, Brautigam CA, Luo X. SAV1 promotes Hippo kinase activation through antagonizing the PP2A phosphatase STRIPAK. eLife. 2017;6: e30278 10.7554/eLife.30278 29063833 PMC5663475

[59] Fu V, Plouffe SW, Guan K-L. The Hippo pathway in organ development, homeostasis, and regeneration. Curr Opin Cell Biol. 2017;49: 99–107. 10.1016/j.ceb.2017.12.012 29316535 PMC6348871

[60] Dewan A, Salih M, Triggle C, Ding H, Tuana B. Abstract 235: Sarcolemmal Membrane Associated Protein Isoform 1: a Unique Regulator of Glucose Uptake and Metabolism in the Myocardium. Circ Res. 2015;117: A235–A235.

